# Transdermal Drug Delivery Systems: Methods for Enhancing Skin Permeability and Their Evaluation

**DOI:** 10.3390/pharmaceutics17070936

**Published:** 2025-07-20

**Authors:** Elena O. Bakhrushina, Marina M. Shumkova, Yana V. Avdonina, Arsen A. Ananian, Mina Babazadeh, Ghazaleh Pouya, Viktoria V. Grikh, Irina M. Zubareva, Svetlana I. Kosenkova, Ivan I. Krasnyuk, Ivan I. Krasnyuk

**Affiliations:** A.P. Nelyubin Institute of Pharmacy, I.M. Sechenov First Moscow State Medical University (Sechenov University), Moscow 119048, Russia; bakhrushina_e_o@staff.sechenov.ru (E.O.B.); asbutyrate@gmail.com (A.A.A.); babazadekh_m_k@student.sechenov.ru (M.B.); puya_g@student.sechenov.ru (G.P.); grikh_v_v_1@staff.sechenov.ru (V.V.G.); kashlikova_i_m@staff.sechenov.ru (I.M.Z.); kosenkova_s_i@staff.sechenov.ru (S.I.K.); krasnyuk_i_i_1@staff.sechenov.ru (I.I.K.J.); krasnyuk_i_i@staff.sechenov.ru (I.I.K.)

**Keywords:** transdermal systems, skin permeation, permeation enhancers

## Abstract

Transdermal drug delivery (TDD) is an increasingly important non-invasive method for administering active pharmaceutical ingredients (APIs) through the skin barrier, offering advantages such as improved therapeutic efficacy and reduced systemic side effects. As demand increases for patient-friendly and minimally invasive treatment options, TDD has attracted substantial attention in research and clinical practice. This review summarizes recent advances enhancing skin permeability through chemical enhancers (e.g., ethanol, fatty acids, terpenes), physical (e.g., iontophoresis, microneedles, sonophoresis), and nanotechnological methods (e.g., liposomes, ethosomes, solid lipid nanoparticles, and transferosomes). A comprehensive literature analysis, including scientific publications, regulatory guidelines, and patents, was conducted to identify innovative methods and materials used to overcome the barrier properties of the stratum corneum. Special emphasis was placed on in vitro, ex vivo, and in vivo evaluation techniques for such as Franz diffusion cells for assessing drug permeation and skin interactions. The findings highlight the importance of active physical methods, passive nanostructured systems, and chemical penetration enhancers. In conclusion, integrating multiple analytical techniques is essential for the rational design and optimization of effective transdermal drug delivery systems.

## 1. Introduction

### 1.1. Background and Clinical Relevance of Transdermal Drug Delivery

Since ancient times, humans have applied various substances to the skin for both therapeutic and cosmetic purposes. Evidence of this practice can be found in some of the earliest surviving medical texts. For example, the ancient Egyptians used oils, fats, perfumes, and other ingredients to make cosmetic and dermatological products. The Ebers Papyrus, dating from the 16th century B.C., contains numerous formulations for treating various skin conditions. Approximately 1500 years later, the Greek physician Galen—often considered the ‘father of pharmacy’—developed Ceratum Galeni, his most famous formula, which is relatively similar to modern cold cream. The precursors of modern transdermal patches appeared in Ancient China. They included combinations of different medicinal plants, unlike Western-type patches, which contained only one active ingredient. During the era of Paracelsus (1493–1541), nicotine, as a modern transdermal agent, was already used in plasters. The Persian physician Ibn Sina (980–1037) suggested that drugs applied to the skin had both local and systemic effects. However, by the end of the 19th century, confidence in transdermal formulations had waned. For instance, the German pharmacopeia of 1872 listed 28 patch formulations, but by 1883, this number had declined. In the early 20th century, various in vivo studies demonstrated systemic absorption after local application. In 1948, an article was published describing the successful use of 2% nitroglycerin ointment for the treatment of Raynaud’s disease, paving the way for its later application in the treatment of angina pectoris during the 1950s. In 1979, the first transdermal patch, manufactured by ALZA Corporation (Mountain View, CA, USA), containing scopolamine, was introduced to the United States of America market for motion sickness treatment. Interestingly, powder of black henbane (a scopolamine-producing plant) was mentioned in the Ebers Papyrus as a remedy for topical or oral application for abdominal discomfort [1].

Transdermal drug delivery (TDD) is a method of administering active pharmaceutical ingredients (APIs) by penetrating intact skin and delivering them at a controlled rate to the target organ via systemic circulation. Unlike topical dosage forms, which only allow the accumulation of APIs on the skin surface or in its upper layers, TDD enables the achievement of systemic action. Nowadays, dosage forms such as gels, creams, ointments, suspensions, emulsions, sprays, and transdermal patches are used for TDD [2].

This delivery system offers a range of advantages over oral and injectable routes, such as prolonged therapeutic effect by maintaining stable drug concentration and minimizing significant fluctuations, bypassing the gastrointestinal tract and first-pass metabolism, allowing direct entry into the systemic circulation (comparable to intravenous administration), providing sustained and controlled release, ease of use, reduced frequency of administration, absence of pain, and improved patient compliance [3].

**Figure 1:**
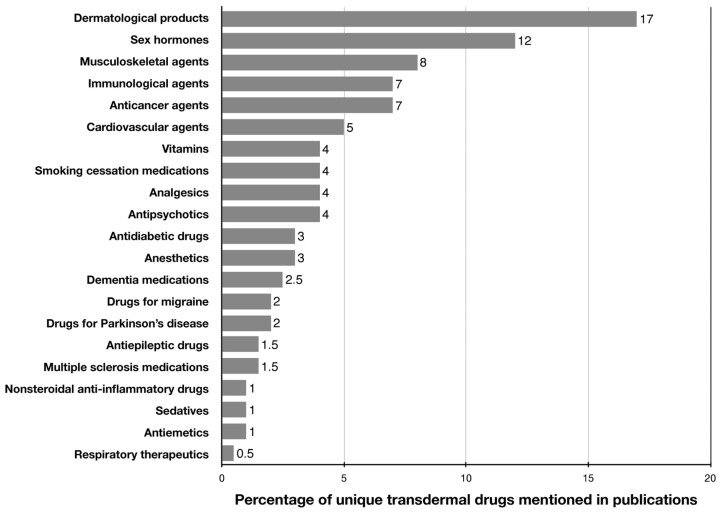
Pharmacological categories of medicines used for TDD.

TDD is used to deliver substances from different pharmacological and chemical groups. To visualize the scope of transdermal systems across therapeutic areas, we performed a literature-based frequency analysis. Publications indexed in PubMed from 2010 to 2024 were screened using combinations of the keywords: “transdermal drug delivery system”, “transdermal patch”, “percutaneous absorption”, and relevant pharmacological classes (e.g., analgesics, hormones, antidiabetics, anticancer agents). Duplicate entries, obsolete drugs, and products no longer available on the market were excluded. Studies addressing general delivery systems without a specific active pharmaceutical ingredient were also not included. Furthermore, drugs that do not exhibit or are not intended for transdermal activity were excluded from the analysis. The relative frequency of eligible studies for each drug class was normalized to 100% (Figure 1). It has been widely applied in delivering therapeutic agents for the treatment of nervous system disorders. For example, Vora and Banga (2022) developed a transdermal patch for delivering olanzapine, a drug indicated for schizophrenia and bipolar disorder [4]. Several studies have explored the use of transdermal cannabidiol for the treatment of peripheral neuropathy in the lower extremities, developmental and epileptic encephalopathies, and Martin-Bell syndrome [5,6,7]. Additionally, transdermal delivery can be used for the administration of macromolecules. For instance, studies by Steffen et al. (2013) and Eypper et al. (2013) described the use of TDD for immunization against travelers’ diarrhea and listeriosis, respectively [8,9]. The study by Kim et al. (2018) demonstrated the successful use of *D. farinae* extract via transdermal delivery for allergen-specific immunotherapy in atopic dermatitis [10]. Walczak et al. (2013) employed myelin peptides for the transdermal treatment of multiple sclerosis [11]. A study by Iannitti et al. (2014) described the process of transdermal delivery of lidocaine with botulinum toxin A for treating primary palmar, plantar, and axillary hyperhidrosis [12]. Drug delivery through the skin has also been employed for the treatment of genitourinary disorders. For instance, Mehrsai et al. (2013) administered verapamil and dexamethasone transdermally to treat Peyronie’s disease [13]. Roth et al. (2014) studied a gel based on segesterone acetate for male contraception [14]. TDD can also be used for the delivery of vitamins and trace elements. Kapoor et al. (2018) developed a system for delivering folic acid into the systemic circulation through the skin, aiming to treat micronutrient deficiencies [15].

### 1.2. Limitations on the Use of TDD

**Figure 2:**
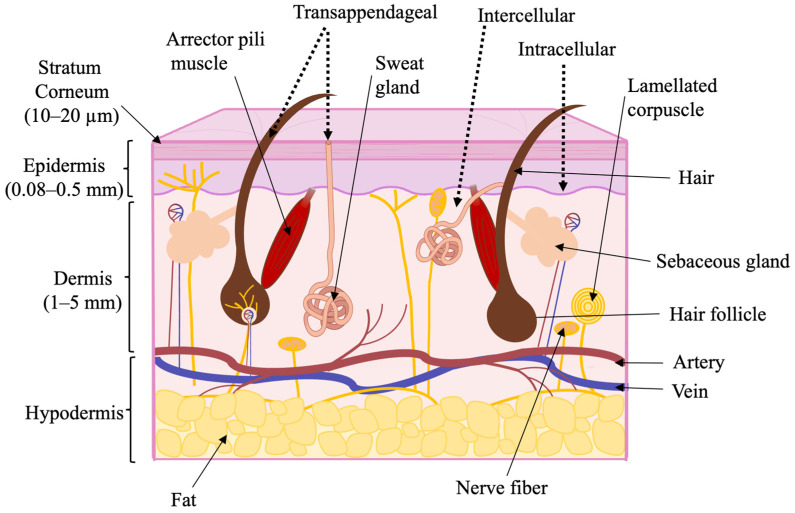
Illustration of human skin layers and principal pathways for transdermal drug penetration.

Despite its advantages, TDD, like other administration routes, has certain limitations. Effective transdermal delivery through the skin remains a significant challenge due to the stratum corneum’s formidable barrier function, the epidermis’s outermost layer [3] (Figure 2). This layer, consisting of corneocytes embedded in a lipid matrix, limits the passive diffusion of most drug molecules, especially those with high molecular weight or poor lipophilicity [1].

To overcome this barrier, various improvement strategies have been developed, which can be broadly divided into three categories: chemical enhancers (e.g., ethanol, fatty acids, surfactants), physical methods (e.g., microneedles, iontophoresis, electroporation), and nanotechnological approaches (e.g., liposomes, ethosomes, solid lipid nanoparticles, and transferosomes). Each of these methods works through different mechanisms, ranging from lipid disruption and increased hydration [15] to the mechanical creation of microchannels or carrier-mediated transport [16,17].

### 1.3. Skin as a Biological Barrier

Human skin is composed of three layers: the epidermis, dermis, and hypodermis. Furthermore, the epidermis is divided into four sublayers: the stratum basale, stratum spinosum, stratum granulosum, and stratum corneum. The stratum basale consists of progenitor cells—undifferentiated keratinocytes responsible for the continuous renewal of the epidermis. The stratum spinosum and stratum granulosum are composed of nucleated keratinocytes and form approximately 15 to 20 cell layers. As keratinocytes lose their nuclei and intracellular organelles during differentiation, keratin progressively accumulates, leading to the formation of the stratum corneum. The structure of the stratum corneum resembles a brick wall, composed of corneocytes embedded in a lipid matrix, about 10–20 μm thick. This layer is essential to the skin’s barrier function, significantly restricting the permeation of hydrophilic and high-molecular-weight molecules. Beneath the epidermis lies the dermis, a fully vascularized layer that contains sweat glands, hair follicles, nerve endings, and lymphatic vessels. The extensive vascular network within the dermis helps establish the concentration gradient necessary for transdermal drug delivery. Below the dermis is the hypodermis, which is primarily composed of adipose tissue. This layer functions in fat storage, cushioning, and thermal insulation. Effective TDD requires drug penetration through the stratum corneum and diffusion across all skin layers (Figure 2) [18,19,20,21,22,23].

There are three primary pathways for drug penetration through the skin: transfollicular, transepidermal, and transglandular (Figure 2). Since skin appendages account for only about 1% of the total skin surface area, the transfollicular and transglandular routes contribute minimally to maintaining constant systemic drug levels. However, skin appendages can facilitate a rapid onset of therapeutic action and serve as “gateways” for ions, large polar molecules, polymers, and colloidal particles. It is widely accepted that intercellular diffusion is the predominant route for most small, uncharged molecules within the transepidermal pathway, whereas transport between corneocytes may dominate for hydrophilic compounds. The lipid bilayers between keratinocytes in the stratum corneum act as a major barrier, limiting the rate of percutaneous absorption [21].

### 1.4. The Influence of Physical and Chemical Properties on Skin Permeability

Transdermal drug delivery is also affected by various physiological parameters such as moisture content, functional state, anatomical site, age, metabolic rate, and body temperature, among others [24,25,26,27].

Molecule size is another important parameter to consider during development. Numerous studies show that molecules with a molecular weight of 500 Da or less can effectively penetrate the skin barrier [28,29,30]. Additionally, several physicochemical properties significantly influence skin permeation, including aqueous solubility, lipophilicity, melting point, pH of the saturated aqueous solution, dosage, and the absence of dermal metabolism (Table 1) [4,24,27,29,30].

## 2. Methods for Enhancing Skin Permeability

Since most drug molecules do not inherently meet the criteria for effective transdermal delivery, various strategies have been developed to enhance skin permeability (Table 2). These approaches fall into three major categories: chemical, physical, and nanotechnological methods.

The disadvantages of chemical enhancers are that they can be relatively costly to produce; however, they can be synthesized on a mass scale and are easy to design, so they can be easily applied to the manufacturing process of diverse formulations like creams, gels, or even patches [31]. However, their safety profile acts as the main limitation of their use, since various potent chemical enhancers induce cytotoxicity, skin irritation, and allergic reactions, especially when they can penetrate the corneum and reach the viable epidermal cells [31,32]. Despite these concerns, the simplicity of chemical enhancers makes them highly scalable, and their compatibility with high-throughput screening platforms such as INSIGHT allows the rapid identification of synergistic combinations that work in humans [31,33]. However, finding the delicate balance between efficacy and skin tolerability often requires extensive safety testing, which can mean increased development costs [26,34].

Physical enhancement methods deliver drugs effectively with less pain, especially the microneedle, which has good patient compliance and only mild and temporary skin irritation [35]. However, some techniques, like electroporation and sonophoresis, can be accompanied by irritation, skin barrier impairment, or reversible pore formation, suggesting a concern for safety related to procedural intensity or duration of exposure [36]. Scalability is a key challenge, since the complex microfabrication process is involved, notably for microneedles and portable sonophoresis devices, which are not yet widely approved by the clinical authorities [16]. Moreover, the dependence on devices and advanced manufacturing results that, when compared to chemical methods, production costs become greater, thus not allowing an extended use without investment in this respect [31,37,38].

Enhancement strategies based on nanocarriers are generally considered safe due to the biocompatibility and biodegradability of materials such as liposomes and polymeric nanoparticles, although some types, such as cationic liposomes, may pose a risk to immune cells. Their poor penetration and controlled drug release contribute to a favourable safety profile [38,39]. However, scalability is a major obstacle, especially for liposomes, as consistent, reproducible large-scale production is technically challenging [37,39]. Although nanocarriers can decrease therapeutic doses and increase drug efficacy, the high costs associated with research, development, and regulatory compliance can hinder development [36,37,40].

**Table 2:** Permeation Outcomes.

Enhancer/Method	Example Enhancer(s)	Example of API	Application Form	Improved Permeation Data	Reference
Fatty acid	Oleic acid 5% *w*/*w*	Olanzapine (OLZ)	Suspension applied to stratum corneum in the donor chamber of Franz cell.	3.3-fold increase in enhancement ratio vs. a control formulation of OLZ in propylene glycol	[41]
Essential oil	Perilla-ketone (PEK) (3 and 5% *w*/*v*) from *Perilla frutescens* (L.)	Puerarin	Essential oil used on rat skin in horizontal dual-chamber diffusion cells.	PEK significantly enhanced puerarin penetration, with enhancement ratios of 2.96 ± 0.07 at 3% and 3.39 ± 0.21 at 5% (*w*/*v*).	[42]
Microneedles	Solid microneedle roller (173 ± 26.94 µm micro-channels)	Potassium chloride (KCl)	Aqueous KCl solution applied to porcine skin in Franz diffusion cells after MN pretreatment.	≈10-fold increase in transdermal flux (6.33 ± 18.70 mg cm^−2^ h^−1^ via microneedle-enhanced permeation vs. 0.637 ± 0.02 mg cm^−2^ h^−1^ via passive diffusion)	[17]
Iontophoresis	0.2, 0.5, and 1 mA/cm2 current density iontophoresis via Ag/AgCl electrodes	Tetracycline HCl	Niosomal Tetracycline-HCl gel applied to full-thickness porcine skin in Franz diffusion cells.	At 1 mA cm^−2^, 2.2-fold increase in cumulative permeation versus passive diffusion; ~20% of the loaded dose delivered within the first 30 min	[43]
Liposome	1,2-dioleoyloxy-3-trimethylammoniumpropan (DOTAP)/Span 80/cholesterol liposomes (F-07) incorporated into Carbopol-940 hydrogel	Simvastatin	Optimised liposomal gel, applied topically to full-thickness dorsal-skin wounds in rats.	The optimized liposome gel achieved a 97% reduction in wound area after 16 days, compared to the posistive control gel with 59.67% reduction rate.	[44]
Ethosome	Soybean-lecithin ethosomes (30% *v*/*v* ethanol) dispersed in Carbomer-940 gel	Huperzine A	Ethosomal gel applied to full-thickness mouse abdominal skin in Franz diffusion cells.	The Huperzine A ethosome gel showed enhanced permeation, with a Q24 of 40.99 ± 4.83 μg/cm^2^, outperforming the ordinary gel (21.49 ± 1.99 μg/cm^2^) and cream (16.80 ± 1.57 μg/cm^2^) (*p* < 0.01).	[45]

### 2.1. Chemical Methods of Permeability Enhancement

Chemical method of enhancing permeability involves the use of agents—known as penetrators—that increase skin permeability. According to the database published by Vasyuchenko et al. (2021), more than 600 chemical compounds are currently classified as chemical penetrators [46]. These substances can be classified by chemical structure: water, hydrocarbons, alcohols, acids, amines, amides, fatty acid esters, surfactants, terpenes, essential oils, and sulfoxides. The characteristics of these substances are described in detail below and are also compared in Table 3.

#### 2.1.1. Water

Water has long been known as a natural penetration enhancer to the skin; stratum corneum (SC) hydration condition is crucial in the enhancement of skin content penetration [31,46]. In general, the higher SC hydration, the greater is the transdermal drug passage [26]. Even though the exact mechanism is not known, water forms pools in the lipid bilayers, the phase separation of lipids and water, which may alter the structural integrity of the SC [46].

#### 2.1.2. Hydrocarbons

Hydrocarbons such as alkanes, alkenes, squalane, squalene, and mineral oil are compounds that may enhance the percutaneous penetration of a drug. Their mechanism of action is due to the disruption of the lipid bilayer and the loosening of the tight structure of SC. For instance, alkanes containing 9–10 carbon atoms showed the highest enhancement for drugs such as propranolol and diazepam, while shorter-chain alkanes were more effective in enhancing caffeine penetration [31].

#### 2.1.3. Alcohols

Alcohols are widely used as carriers, solvents, or enhancers for the penetration of drugs in transdermal preparations. The major mechanisms of action are lipid and protein extraction from the SC, leading to swelling of the SC and increasing drug solubility in the formulation or distribution across the skin [31]. Ethanol is frequently employed as a cosolvent to enhance drug permeation through SC by perturbing SC lipid domains and to enhance the local concentration of the active agent. Ethanol, as opposed to other alcohols, is generally well tolerated in everyday use, as its impact on keratinocytes and SC enzymatic activity is tolerable [34,47].

#### 2.1.4. Fatty Acids

Long-chain fatty acids, especially oleic acid, are one of the most widely investigated skin penetration enhancers [26]. Lipid disruption is a primary mechanism used in many chemical and nanotechnological enhancers. The SC, composed of corneocytes embedded in a lipid matrix (often described as a “brick and mortar” model), provides a formidable barrier to most drugs. The mechanism of action of fatty acids involves disruption of the structured lipid domains of the SC to increase drug permeation and facilitate the formation of lipophilic drug–fatty acid complexes [31]. The permeability-increasing effect appears to be greater with unsaturation and cis- than trans-unsaturated fatty acids, the latter causing minor lipid-disruption. Moreover, an increased distance between the carboxyl group and the double bond has been shown to enhance drug permeation. Often, fatty acids are mixed with co-solvents to produce synergistic actions [26,27,34,46].

#### 2.1.5. Esters of Fatty Acids

Fatty acid esters are commonly used as permeability enhancers, with isopropyl myristat being a popular example. Their mechanism of action is interpreted to be by disordering SC lipids. They also act by increasing the membrane fluidity, thereby allowing the penetration of drugs and improv improving of solubility in the SC [31,48,49].

#### 2.1.6. Amines

Amines have commonly been employed for improved percutaneous penetration of drugs. They are believed to work on the disruption of lipid bilayer structure or enhance local active compound distribution among the skin layers [31].

#### 2.1.7. Amides

The amide classes, both cyclic and acyclic, are a large group of penetration enhancers, and Azone was the first compound synthesized specifically for this application [26,31]. Azone and its analogs have been widely investigated. Azone is active at very low concentrations and acts by penetrating the lipid bilayer and disordering its structure [46,49]. Another subgroup of amides, pyrrolidones, promotes drug permeation by their intercalation in the lipid matrix, changing lipid fluidity, which reduces the barrier to drug diffusion. Urea, as well as derivatives in this class, generally work by degrading skin lipids [31,46,49].

#### 2.1.8. Surfactants

A wide range of surfactants, including anionic, cationic, zwitterionic, and nonionic types, are skin permeability enhancers. Their activity depends on factors such as hydrophilic-lipophilic balance, charge, and lipid tail length [31,46]. Surfactants can act by binding to or denaturing skin proteins, solubilizing or disrupting intercellular lipids, penetrating the SC, or interacting with corneocytes [46].

#### 2.1.9. Terpenes

Terpenes, which are natural constituents of essential oils, have been employed as a skin penetration enhancer agent level. Generally regarded as safe, since minimal, transient irritation has been reported [49]. Their mechanism of action is essentially the disruption of the lipid organization of the SC with subsequent drug penetration [49]. For hydrophilic drugs, it has been recommended that terpenes containing polar functional groups are more active, while hydrocarbon terpenes are suitable for improving the absorption of lipophilic compounds [50].

#### 2.1.10. Essential Oils

Essential oils are focused on complex volatile compounds obtained from aromatic plants, and they are commonly known for their potential to improve skin penetration [50]. Their main mode of action is the disorganization of the closely packed intercellular lipids from the SC so that its permeability increases. They may also meet structural proteins of the SC, which lead to conformational changes and, in turn, favor drug penetration. As reported to be safe, low-molecular-weight essential oils are metabolized rapidly, not accumulated in the body, and effectively excreted after topical application [32,50].

#### 2.1.11. Sulfoxides

Sulfoxides, especially dimethyl sulfoxide (DMSO), are one of the first and most well-studied skin penetration enhancers [31]. DMSO serves as a strong aprotic solvent that interacts with hydrogen bonds rather than water, allowing fast onset of action [31]. Its enhancing effects are attributed to disturbing skin lipids, binding with keratin, and changing the aqueous microenvironment of the SC [46]. DMSO also extracts lipids and creates water channels, which increases SC permeability [26]. Nevertheless, extreme enhancement generally demands high concentrations that can be harmful to result in erythema, blistering, peeling, urticaria, tingling, burning, and protein denaturation [31,48].

**Table 3:** Percutaneous Penetration Enhancers.

Type	Mechanism	Advantages	Disadvantages	Examples	Reference
Water	Swelling of the stratum corneum and loosening of its compact structure facilitate drug permeation	The most natural penetrator increases penetration of both hydrophobic and hydrophilic drugs	Limited action	Water	[31,46]
Hydrocarbons	Disruption of the lipid structure of the stratum corneum	Good solvents for lipophilic drugs; occlusive properties improve skin hydration	Low enhancement for hydrophilic drugs; greasy residue may affect patient compliance	Alkanes, alkenes, halogenated alkanes, squalane, squalene, mineral oil	[31]
Alcohols	Lipid and protein extraction; Swelling of the stratum corneum; Improving the distribution of drugs in the skin; Drug supersaturation	Increasing diffusion rate due to drug supersaturation; Cyclic polyols can interact with biological barriers; The use of propylene glycols minimizes drug contact time with tissues	Isopropanol and n-propanol cause significant disruption of the stratum corneum and keratinocytes; Fatty alcohols increase transepidermal water loss,	Ethanol, glycerol, fatty alcohols, cyclic polyols, Isopropyl alcohol	[31,34,47,51]
Fatty acids	Disruption of lipid structures of the stratum corneum; Enhanced spreading of drugs in the stratum corneum; Formation of lipophilic complexes with drugs	Good skin compatibility; effective for both hydrophilic and lipophilic drugs, low cost	Less efficient trans-configurations and unsaturated compounds	Linoleic, Lauric, oleic, caprylic, Palmitoleic, and other fatty acids	[26,27,30]
Esters of fatty acids	Disruption of the lipid organization within the stratum corneum	Enhanced transdermal penetration of a wide range of drugs has been found	At high concentrations, Transcutol^®^ can dehydrate the stratum corneum, thereby reducing transdermal drug penetration	Isopropyl myristate, Isopropyl palmitate, Transcutol^®^, Ethyl oleate	[31,48,52]
Amines	Improving the distribution of drugs in the skin; Separation of the lamellar lipid bilayers of the stratum corneum	Disrupt lipid packing; enhance delivery of a wide range of actives	Potential for irritation or allergic response with prolonged use	Primary, secondary and tertiary, cyclic and acyclic amines	[31]
Amides	Reducing the diffusion resistance of the drug substance in the stratum corneum; Integrating into the region of the lipid bilayer; Increased fluidity of stratum corneum lipids; Disruption of lipid structures of the stratum corneum	Enhance the penetration of hydrophilic and hydrophobic compounds and some peptides	Adverse effects associated with the use of pyrrolidones; Insufficient chemical stability of urea	Azone (laurocapram), pyrrolidone, urea	[31,46]
Surfactants	Denaturation or binding to skin proteins; increased fluidity of intercellular lipids in the stratum corneum; direct penetration through the stratum corneum or interaction with corneocytes	Improve solubilization and diffusion of drugs; widely used in formulations, a broad spectrum of surfactants is commercially available on the market	Cationic surfactants cause dermal irritation	Anionic (Sodium lauryl sulfate), cationic amines, alkyl imidazolines, alkoxylated amines and quaternary ammonium compounds), zwitter-ionic, non-ionic, Cetyltrimethylammonium bromide	[31,46]
Terpenes	Influence on the polar hydrophilic end of the lipid bilayer; Disruption of the hydrogen bonding network; Formation of new polar channels in the skin	Temporary and relatively low skin irritation; Higher penetration characteristics; The combination of terpenes improves the penetration effect; Enhanced membrane lipid fluidity	Can cause mild to moderate irritation at high concentrations	Oxygen-containing sesquiterpenes, menthol, Eugenol, 1,4-cineole, 1,8-cineole, Thymol, Limonene	[49,50]
Essential oils	Disintegration of highly ordered intercellular lipid structure between corneocytes in the stratum corneum; Interaction with intercellular protein leading to conformational modifications	Safety; Quickly metabolized; Not accumulated in the body; Quickly excreted from the body after application to the skin; Enhanced drug distribution in the stratum corneum”	Potential for allergic reactions with repeated use	Perilla-ketone (PEK), Peppermint Oil, Turpentine Oil	[32,50,52]
Sulfoxides	Formation of solvent-filled free spaces in the stratum corneum	High-efficacy	Locally irritating effect	Dimethyl sulfoxide	[31,48]

### 2.2. Physical Methods of Permeability Enhancement

Physical methods for enhancing permeability are techniques based on the use of electric current or mechanical impact on the skin. Among them are: (1) iontophoresis, (2) sonophoresis, (3) microneedles, (4) elongated microparticles, (5) electroporation, and (6) needle-free jet injection. The characteristics of these methods are described in detail below and are also compared in Table 4.

#### 2.2.1. Iontophoresis

Iontophoresis is a method of transdermal drug delivery that uses an electrical current. A charged molecule is placed on the skin with an electrode of the same charge [35,53,54,55]. Direct current drives the drug into the skin, where it is absorbed into the bloodstream and deeper tissues [53].

A key advantage of iontophoresis is its ability to precisely control the rate and extent of delivery. This allows for dose adjustment by adjusting the duration, intensity, and profile of the current. That control enables the administration of therapeutic drug amounts without an infusion pump [56].

Iontophoresis is considered medically necessary for clinical applications like administering local anesthesia before a venipuncture or dermatological procedure [35,53,55] and short-term (less than 24 h) management of acute postoperative pain in adult patients requiring opioid analgesia using fentanyl [35,53].

Some iontophoretic patch systems have been approved by the U.S. Food and Drug Administration, including LidoSite™ for rapid local anesthesia [35,55] and Ionsys™ for systemic fentanyl delivery [35]. This method is particularly suited for hydrophilic and ionizable molecules [35], including peptides and proteins [33,35,37,38]. Studies have demonstrated its potential for the noninvasive delivery of insulin [33,35], human basic fibroblast growth factor [35,38], and a variety of NSAIDs and corticosteroids for musculoskeletal conditions [35,53].

Despite its advantages, the use of iontophoresis for other medical indications is investigational [53]. Limitations include the potential for skin irritation, a burning sensation, and burns, which can occur due to local heating and pH changes at the electrodes [54]. The effectiveness of delivery can also be limited by factors such as low-level currents, which restrict the transport efficiency of large molecules [54], and the accumulation of H^+^ and OH^−^ ions at the electrodes. This can cause insufficient drug delivery if the current density or duration is limited [33]. Furthermore, excess free ions in the formulation can compete with the API, reducing iontophoretic transport [38]. Traditionally, iontophoresis required an external device, but newer approaches are exploring portable, user-friendly systems [33]. Iontophoresis can be combined with techniques such as electroporation or microneedles to enhance transdermal drug delivery synergistically [53,54,57].

#### 2.2.2. Sonophoresis

Sonophoresis is a transdermal drug delivery technique that employs ultrasound to enhance solute penetration into and across the skin [38,53,58]. This method operates by applying a longitudinal sound wave with frequencies ranging from 20 kHz to 16 MHz [37,54,58].

The primary mechanism underlying sonophoresis is cavitation, which involves the formation, expansion, contraction, and collapse of gaseous bubbles in a liquid medium, such as the coupling medium or the intercellular lipids within the skin [37,38,58]. This mechanical action can create aqueous channels or microchannels in the stratum corneum, the outermost layer of the skin, thereby increasing its permeability [37,53,58]. Although thermal effects due to ultrasound energy absorption can also increase skin permeability, cavitation is considered the prime mechanism of action [58].

Sonophoresis is particularly suited for delivering hydrophilic molecules and macromolecules transdermally, which is generally poor. Studies have demonstrated the efficacy of sonophoresis in delivering a wide range of therapeutic agents, including local anesthetics such as lidocaine, opioids such as fentanyl, nonsteroidal anti-inflammatory drugs (NSAIDs) such as ketoprofen, and large molecules such as insulin and heparin [33,38,53].

#### 2.2.3. Microneedles

Microneedles are a minimally invasive transdermal drug delivery system that employs micrometer-scale needles to enhance the penetration of solutes into and across the skin [35,54]. These microstructures are engineered to painlessly breach the outermost layer of the skin, the stratum corneum, and the epidermis without reaching the pain-sensitive nerve endings in the dermis. This process creates microchannels that significantly increase skin permeability [35,54].

These substances exist in a variety of forms, including solid, drug-coated, dissolving, hollow, and hydrogel-forming types. These different forms possess varying delivery mechanisms [35,54].

The primary advantages of microneedles include their patient-friendly nature (reduced pain relative to conventional hypodermic injections) and their potential for self-administration, which can significantly improve patient compliance [16,35,54].

These patches have been found to be particularly effective for the transdermal delivery of a wide array of drugs, including large hydrophilic molecules, macromolecules, peptides, and proteins such as insulin and vaccines. This is a notable advantage over passive transdermal transport, which has proven challenging in this regard [33,54]. The combination of microneedles with other enhancement techniques, such as iontophoresis, has been demonstrated to yield supplementary enhancement of transdermal delivery [54]. In addition to their therapeutic applications, microneedles are being explored as potential platforms for point-of-care diagnostics, with the objective of enabling painless withdrawal of biological fluids [16].

Despite their considerable promise, microneedles face several limitations and challenges that hinder widespread clinical adoption. These include concerns about their mechanical robustness and the skin’s elastic properties, which can lead to incomplete penetration or breakage of the needles [54]. The potential adverse effects of this treatment include skin irritation, erythema, and edema. However, these effects are typically mild and short-lived [54]. The cost of fabrication and the necessity for aseptic manufacturing persist as substantial economic factors [16,37]. Furthermore, challenges arise in the formulation process due to limitations in the drug loading capacity of specific microneedle types and the instability of encapsulated drugs during manufacturing [33,37].

#### 2.2.4. Elongated Microparticles

Elongated microparticles (EMPs) represent a minimally invasive physical penetration enhancement technology developed as an alternative to traditional microneedle arrays for transdermal drug delivery [36,38]. These are freely moving microparticles typically around 173.3 ± 100.8 μm in length and 8 μm in diameter, fabricated from materials like silica oxide filament [36]. EMPs enhance drug penetration by being mixed with a drug formulation and applied topically via gentle rubbing on the skin, allowing them to painlessly penetrate the outermost layers to the dermal-epidermal junction (DEJ), primarily within the epidermis [36,38]. This mechanical action creates transient microchannels and a zone of mechanical disruption, which A dark halo may manifest around the EMPs, signifying the transient rupture of cells. EMPs can be dry-coated with active pharmaceutical ingredients using methods such as air or freeze-drying, enabling controlled release kinetics, including sustained or burst release, depending on the coating strategy (e.g., alginate cross-linking for sustained release) [36,38].

A significant advantage of EMPs is their flexibility, as they can be applied over irregular skin surfaces of various sizes, and their unbound nature circumvents the “bed-of-nails” effect encountered with fixed microneedle arrays, which is particularly beneficial for topically treating diseased or aged skin with heterogeneous morphology [36,38]. This technology has demonstrated its ability to enhance the cutaneous delivery of a diverse array of payloads, including small molecules (such as 6-carboxyfluorescein, sodium fluorescein, diclofenac, nicotinamide, and aminolevulinic acid), as well as larger hydrophilic molecules like peptides, proteins, vaccines, nutraceuticals, cosmetics, and other nano-/micro-particles [36,38]. EMPs are designed to naturally exit the skin via epidermal turnover after application [38]. While generally minimally invasive, the application may cause temporary erythema and mild irritation that typically resolves within 24 h [36,38]. Further research is necessary to fully assess their safety and tolerability in inflammatory skin diseases, and their efficacy is influenced by the viscosity of the drug formulation, making them most suitable for low-viscosity creams, gels, and lotions [36].

#### 2.2.5. Electroporation

Electroporation is a novel transdermal drug delivery technique that utilizes short, high-voltage electrical pulses to temporarily and noninvasively increase skin permeability, specifically the outermost layer known as the stratum corneum [37,57,59]. This process creates reversible channels or transient pores within the SC’s lipid bilayers, significantly enhancing the transdermal diffusion of active compounds [33,37,57,59]. The primary mechanism involves the high-voltage pulsed electric field disturbing the structure of the stratum corneum [37,57]. Unlike iontophoresis, which uses a sustained low-level current, electroporation relies on short, high-voltage energy [33,54].

A key advantage of electroporation is its ability to enhance the transdermal delivery of a diverse array of molecules, including large hydrophilic drugs, macromolecules, peptides, proteins, and even genetic material like DNA, which are otherwise difficult to transport across the skin [33,37,57,59]. The efficiency of electroporation can be adjusted by changing parameters such as pulse voltage, duration, amount, and speed of stimulation, allowing control of transdermal penetration [33,37,57,60]. Studies have shown that the pores created by electroporation can persist for at least 2 h, with effects lasting over 12 h before the skin gradually restores its barrier properties [37,57]. Increased electroporation power generally leads to higher cumulative drug permeation, indicating that pulse intensity is often more critical than duration for transdermal permeation [57].

Electroporation can be applied for various medical indications, including delivering local anesthetics like lidocaine, opioids such as fentanyl, and crucial large molecules like insulin and vaccines [33,37,54,57,59,60].

While electroporation significantly enhances transdermal transport, especially for charged molecules [33], a notable limitation is the potential for pain and muscle stimulation due to the high-voltage pulses affecting subdermal structures [33,54]. Skin irritation, erythema, and edema may also occur, although these are typically transient [60]. The use of microelectrode arrays and optimizing device design are ongoing efforts to mitigate these side effects [33,54]. Despite its promise, widespread clinical adoption of electroporation for general drug delivery has been hindered by these challenges and the cost and cumbersomeness of some devices [33,59]. However, it can be combined with other techniques, such as iontophoresis, to achieve synergistic improvements in drug efficiency [57].

#### 2.2.6. Needle-Free Jet Injection

Needle-free jet injection is a technique that delivers a high-speed stream of liquid medicine (60–140 m/s) to penetrate the epidermis and spread into subcutaneous tissue, bypassing the use of conventional needles [33,35]. This method uses a power source, such as compressed gas or a spring, to generate a high-velocity jet from a nozzle, typically with an orifice diameter of 50 to 360 µm. Rather than being distributed as a bolus, the fluid stream disperses in an approximately hemispherical pattern within the skin, following the path of least resistance [35]. The depth of penetration (intradermal, subcutaneous, or intramuscular) and delivery efficiency are influenced by factors like injection pressure, initial liquid velocity, nozzle diameter, and distance from the skin, as well as the mechanical properties of the skin itself [33,35].

Key advantages of jet injection include its needle-free nature, which leads to less injury and pain compared to traditional syringes, and its potential for improved penetration and absorption due to drug dispersion over a larger skin area [33,54]. It also offers a faster onset of pharmacological effect for some drugs, such as insulin, which reduces blood glucose levels within one hour, and can mimic endogenous insulin secretion more closely [33,54]. Furthermore, needle-free devices lower concerns about accidental needle sticks and hazardous sharps waste [35].

However, jet injection faces several limitations and challenges. It can cause adverse reactions such as bruising, bleeding, and pain, with some studies indicating that it may cause as much pain as hypodermic needles [33,35,54]. Liquid splashing and cross-contamination are also concerns, especially with multi-use nozzle jet injectors, promoting a shift towards disposable cartridge systems [33,35]. The high cost of jet injection devices and the need for accurate adjustment of drug amount and penetration depth hinder their widespread clinical acceptance [33]. Although the FDA has approved some jet injection devices for transdermal insulin delivery, their adoption remains low due to small injection volumes and inconsistent efficiency, highlighting the ongoing need for optimization in cost, size, and performance [33,54].

**Table 4:** Physical methods of permeability enhancement.

Method	Basis of Method	Advantages	Limitations	Examples	Reference
Iontophoresis	The use of a pair of electrodes placed on the skin to create an electrical potential between the skin surface and capillaries	Does not disturb the structure of the skin; Easy integration of conductive bases with iontophoretic systems	The delivery of negatively charged molecules is hampered; Low amperage can limit transport efficacy; High amperage can increase the risk of skin irritation; Requirement for the use of electrically conductive bases	LidoSite^®^ (lidocaine and epinephrine for anesthesia) IONSYS™ (fentanyl iontophoretic system for acute postoperative)	[53,54,55]
Sonophoresis	Use of mechanical force generated by ultrasound that increases skin permeability to drugs through hyperthermia or cavitation	Ability to deliver large and hydrophilic molecules;	High-frequency sonophoresis can cause damage to deep skin tissues; Low-frequency sonophoresis often requires an appropriate environment; The need for sophisticated devices	SonoPrep^®^ (ultrasonic skin permeation system and topical anesthetic kit)	[54,58,61]
Microneedles	Creation of micro-sized pathways for transporting molecules	Less invasive than parenteral routes of administration; Low level of pain and discomfort during use; Self-administration by patients; Ability to deliver macromolecules	Risk of skin irritation or infection; Limited drug loading per microneedle; Requires specialized manufacturing	MicronJet™ (dissolving hyaluronic acid microneedles)	[16,17,62]
Elongated microparticles	When applied, elongated microparticles that may be mixed with the drug pass through the epidermis carrying the drug	Unlimited area of application on the skin; Penetrates primarily into the epidermis, minimizing damage to the dermis; Natural elimination (transepidermal)	More suitable for use with low-viscosity creams, gels, and lotions	Elongated silica microparticles with hyaluronic acid	[36,38,63]
Electroporation	The use of high-voltage electrical pulses of a millisecond or microsecond duration, under which pores are formed in the skin	Facilitates the penetration of hydrophilic macromolecules as well as biomolecules; Formation of reversible pores in the skin	May cause discomfort or skin irritation; Requires electronic devices and energy sources; Not suitable for all drug types	Nanocomposite hydrogel system serving as both drug reservoir and skin electrode for electric-pulse delivery	[57,59,60,64]
Needle-free jet injection	A high-pressure device is used to achieve a high rate of liquid drug injection, allowing the therapeutic agents to penetrate the epidermis and spread into the subcutaneous fatty tissue without the use of a needle.	Ability to deliver macromolecules; Ability to modify the delivery area by flow rate and orifice diameter;	Injection depth may vary; Potential for bruising or local pain; High device cost and maintenance	ZETAJET Needle-Free Injection Therapy System	[33,35,65]

### 2.3. Nanotechnology Methods of Permeability Enhancement

#### 2.3.1. Nanoemulsion and Microemulsion

Nanoemulsions are finely dispersed systems of the oil-in-water, water-in-oil, or bicontinuous type, stabilized by an interfacial film of surfactant molecules with droplet sizes in the nanometer range [36]. Microemulsions are also finely dispersed systems, where one phase is water and the other is oil, and the phases are stabilized by a film of surfactant molecules [66]. According to Shukla et al. (2018), microemulsions are thermodynamically stable, transparent dispersions with droplet sizes typically below 0.1 μm (100 nm), a range in which the terms “microemulsion” and “nanoemulsion” are often used interchangeably in the literature [66]. Microemulsions have many advantages, such as increased skin permeability, good solvation capacity (for both hydrophilic and lipophilic substances), thermodynamic stability, and ease of preparation. These qualities explain the widespread application of microemulsions in transdermal drug delivery systems [67]. One study compared levamisole delivery via a microemulsion-based gel versus a simple gel. The results showed that the microemulsion-based gel had 4.07-fold higher permeability compared to the simple gel [68]. Microemulsions reduce the interfacial tension between the skin surface and the drug and solubilize it. Additionally, lipids or surfactants that may be included in the microemulsion act as a chemical penetrator, dissolving or disrupting the structure of the lipid bilayer of the epidermis. As a result, the barrier function of the stratum corneum is minimized, and channels for drug transfer through the skin are opened [66]. However, high concentrations of surfactants (>20%) are used in the preparation of such microemulsions, which can have an irritating effect on the skin. Consequently, there is growing interest in formulations that minimize surfactant content or utilize natural surfactants. For example, one study developed an oil-in-water microemulsion with a low surfactant concentration, utilizing oleic acid to enhance the transdermal delivery of nifedipine. The results demonstrated that this composition was effective for the transdermal delivery of nifedipine [69]. Another study reports the development of a surfactant-free microemulsion system containing *n*-propanol and eucalyptus oil, which enhances the solubility, stability, and transdermal permeation of all-trans retinoic acid (ATRA). ATRA was spontaneously solubilized in the developed composition due to its interaction with eucalyptus oil. Notably, the solubility of ATRA in this microemulsion was significantly higher than in water or some other microemulsions. Moreover, this composition increased the drug absorption rate [70]. Emulsion systems represent a promising approach in modern biomedicine, offering significant potential for dissolving or encapsulating drug molecules. Additionally, their thermodynamic or kinetic stability contributes to improved shelf life [71].

#### 2.3.2. Liposomes

**Figure 3:**
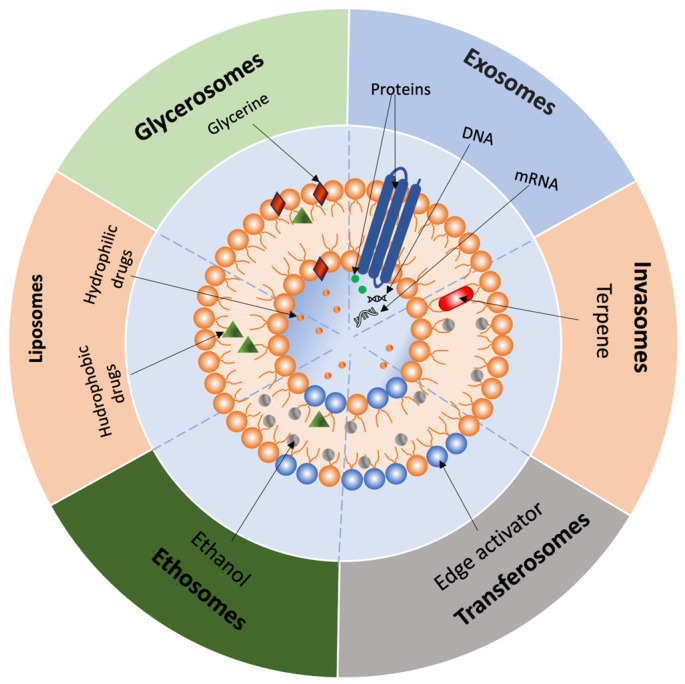
Schematic classification of liposomal nanocarriers and their functional excipients used in transdermal drug delivery.

Liposomes are small, artificially constructed spherical vesicles composed of an aqueous core surrounded by one or more phospholipid bilayers, with the polar head groups oriented toward both the inner and outer aqueous environments (Figure 3) [72]. Due to their structure, liposomes can encapsulate and deliver drug molecules with varying solubility. Hydrophilic molecules can be placed in the aqueous core, lipophilic molecules in the lipid bilayer, and amphiphilic molecules at the interface between the aqueous and lipid bilayers [73]. Glycerophospholipids are the main components of liposomes. These amphiphilic molecules are composed of glycerol linked to a phosphate group and two chains of saturated or unsaturated fatty acids [74]. Glycerophospholipids can be classified as natural and synthetic. Examples include phosphatidic acid (PA), phosphatidylethanolamine (PEA), phosphatidylcholine (PC), phosphatidylglycerol (PG), phosphatidylinositol (PI), and phosphatidylserine (PS), which are natural glycerophospholipids [75]. Among them, PC and PEA are primarily used in liposome formulation. In addition to phospholipids, cholesterol, polyethylene glycol, propylene glycol, and chitosan are used to improve the stability of liposomes [76]. The most common methods for manufacturing liposomes include: (1) thin-film hydration, (2) reverse-phase evaporation, (3) solvent injection techniques, (4) detergent removal, (5) dehydration-rehydration, (6) pH jump, (7) hydration in a compact layer of colloidal particles, and (8) freeze-thawing [77]. Traditional liposomes have several limitations, such as a short half-life, rapid clearance by the reticuloendothelial system, and instability in plasma [78]. To address these challenges, second-generation liposomes have been developed, including stealth, PEGylated, and ligand-modified liposomes [39,40,78,79,80].

#### 2.3.3. Invasomes

Invasomes are a novel class of liposomes incorporating terpenes as natural penetrators. They consist of PC, ethanol, and a standard mixture of terpenes (cineole:citral:d-limonene in a (45:45:10 *v*/*v*) ratio). Due to the synergistic effect of the vesicles, ethanol, and terpenes, invasomes enhance the penetration of various drug substances through the skin. Ethanol interacts with stratum corneum lipids, leading to lipid fluidization and disruption of its densely packed structure. Terpenes act as penetration enhancers by disrupting the tightly packed structure of the lipid bilayers and interacting with intercellular proteins in the stratum corneum (Figure 3). Invasomes have demonstrated a proven effect in enhancing the penetration of drug substances such as curcumin, isradipine, nimesulide, avanafil, and luteolin [81,82]. For example, in a study conducted by A. Zaffar et al., a luteolin-loaded invasomal hydrogel provided approximately 2.8 times higher skin flux and about 3 times greater permeability coefficient compared to a conventional luteolin gel. This resulted in roughly a 2.4-fold increase in systemic bioavailability compared to oral administration. This enhanced delivery method resulted in substantial improvements in pharmacodynamic outcomes. Specifically, the invasomal luteolin gel demonstrated a significant reduction in inflammatory paw edema compared to the non-invasomal gel. In a study by El-Kayal et al., the efficacy of a luteolin-loaded invasomal gel for the treatment of psoriasis was investigated in comparison with nanostructured lipid carriers, including liposomes. The invasomal gel demonstrated approximately 2.1 times higher drug accumulation in the stratum corneum and 5.5 times greater deposition in the epidermis and dermis compared to the control suspension, as well as 1.5 times deeper skin penetration than liposomal formulations. While the in vitro anti-inflammatory activity of invasomes and liposomes was comparable, in an in vivo rat model of psoriasis, invasomes provided a significantly greater reduction in inflammation and skin lesions compared to both the suspension and the liposomes. These differences suggest that invasomes, owing to their ultraflexible structure and terpene content, offer an advantage over conventional liposomes for transdermal delivery of active compounds [83].

#### 2.3.4. Transferosomes

Transferosomes are liposome derivatives. They represent lipid-based vesicular carriers that, unlike liposomes, are elastic, ultra-deformable, and stress-resistant. Transferosomes consist of four key components: (1) phospholipids (e.g., PC), (2) surfactants (10–25%), (3) ethanol (up to 10%), and (4) water. Their deformable properties enable them to penetrate through skin membranes, allowing for increased vesicle penetration without measurable losses. They contain both organic and aqueous phases in their structure, allowing them to accommodate drug molecules with a wide range of solubility (Figure 3) [84]. Recent studies have further highlighted the advantages of transfersomes in enhancing transdermal drug delivery, particularly in terms of drug loading, penetration efficiency, and pharmacological performance. The study by Khan et al. demonstrated that a transfersomal gel containing meloxicam and dexamethasone exhibited high drug encapsulation, sustained release, and significantly enhanced skin permeability compared to conventional formulations. In an ex vivo model, drug penetration from the gel reached up to 86%, whereas plain suspensions achieved less than 27%. The gel also showed good stability and caused no skin irritation, confirming the potential of transfersomes as an effective system for transdermal delivery of anti-inflammatory agents [85].

#### 2.3.5. Ethosomes

Ethosomes are a specific type of transferosomes distinguished by a higher ethanol content in their composition [86]. It is assumed that the mechanism of ethosome permeation into the skin is due to ethanol and phospholipids. Ethanol interacts with the lipids of the stratum corneum, leading to lipid fluidization and disruption of its densely packed structure. Thus, the soft vesicles of the ethosomal system penetrate the altered structure of the stratum corneum. Drug release occurs when ethosomal vesicles fuse with cell membranes in the deeper skin layers (Figure 3) [86]. Recent findings demonstrate that ethosomes provide superior transdermal delivery compared to transferosomes. In a comparative study using sinapic acid (SA) as a model compound, ethosomal hydrogel achieved significantly higher skin permeation (66.5 ± 7.3 µg/cm^2^) than transferosomal (53.2 ± 1.5 µg/cm^2^) and plain hydrogel (12.3 ± 1.4 µg/cm^2^). This enhancement is attributed to the high ethanol content and flexibility of ethosomal vesicles, facilitating deeper penetration through the stratum corneum [87].

#### 2.3.6. Glycerosomes

Glycerosomes are innovative, flexible vesicular carriers for transdermal delivery, characterized by a high glycerin content (10–30%, *v*/*v*). They outperform liposomes in terms of encapsulation efficacy [88,89], shelf life, penetration into the deeper layers of the skin, and ease of scaling up their production technology without high temperatures [89]. Glycerin is a non-toxic and non-irritating excipient for our skin, making it suitable for safe use in new pharmaceutical forms. The increased triatomic alcohol content (20–30%, *v*/*v*) in glycerosomes enhances the fluidity of the phospholipid bilayer compared to liposomes or glycerosomes with lower glycerin concentrations (10%, *v*/*v*) (Figure 3) [90]. This improved flexibility makes the nanoparticles more deformable, enabling them to penetrate deeper skin layers by compressing and fitting into pores significantly smaller than their size [91,92]. In addition, the penetration mechanism contributes to the ability of glycerin to moisturize the skin and to induce lipid fluidization within skin lipids [93]. Vesicle deformability, evaluated through extrusion studies, has confirmed the elasticity of glycerosomes in drug encapsulation research [89,94,95]. Notably, studies by Mona M. Shahien and Younes N.F. reported that an excessively high concentration of glycerin (40% *v*/*v*) reduces drug encapsulation efficiency [90,92]. The increased viscosity also reduces homogeneity of glycerosomes, leading to a higher polydispersity index (PDI) [92]. Besides glycerin, the composition of new carriers typically includes phospholipids, cholesterol, and water [91]. Cholesterol imparts rigidity to the molecules, serves as a barrier in the aqueous phase, enhances the stability of glycerosomes, and maintains membrane integrity, thereby increasing the number of positive charges and preventing nanoparticle agglomeration [96,97]. However, more recent studies have replaced cholesterol with Span60 [98], which offers similar rigidity while resulting in vesicles with a higher zeta potential and smaller size, thereby improving long-term stability [99]. Glycerosomes can facilitate transdermal, pulmonary, and intranasal administration of a wide range of drugs [90]. These nanoparticles have demonstrated effectiveness in enhancing the therapeutic delivery of various active substances such as diclofenac, sulpiride, essential oil of *Melissa officinalis* L., rutin hydrate, atorvastatin, triptolide, itraconazole, extract of *Zingiber officinalis*, a mixture of lincomycin and lauric acid, quetiapine fumarate, lacidipine, lappaconitine, voriconazole, glycyrrhetinic acid, fisetin, and plumbagin [88,90,91,94,95,97,98,100,101,102,103,104,105,106,107,108]. Taken together, these findings suggest that glycerosomes represent a promising strategy for drug delivery across a broad range of medicinal areas. Glycerosomes are not the only option for using glycerin to enhance transdermal delivery. In 1993, the first mention of a compound formed from glycerin and the heavy metal titanium appeared in the Union of Soviet Socialist Republics [109]. The authors aimed to develop a new compound that could increase the transcutaneous permeability of drugs through the skin and mucous membranes, while also reducing the toxicity of administered medications. They confirmed its activity, effectiveness, and non-toxicity at laboratory and preclinical levels through polarographic cells and studies tested on laboratory animals. The compound was registered and received the trade name Tizol^®^. It can penetrate the skin to a depth of 4–9 cm, thereby facilitating drug transport into deeper skin layers and exerting various pharmacological effects, including anti-inflammatory, antimicrobial, protective, dehydrating, anti-edematous, and local analgesic actions [110]. Multiple researchers have confirmed its transcutaneous action when forming a complex with the aquacomplex of titanium glycerosolvate. For instance, Mokhova O.S. and Glukhov A.A. developed a Tizol^®^-based gel formulation for the comprehensive treatment of soft tissue wounds, using oxytocin as the biologically active ingredient. Clinical studies demonstrated that the wound area decreased more rapidly following treatment with the formulated complex [111,112]. In a work by Solvyova A.G. et al., the effectiveness of using Tizol^®^ in combination with other medications for treating burn injuries was evaluated based on liver enzyme activity. The combination of Tizol^®^ with various drugs was shown to enhance therapeutic outcomes by accelerating the normalization of enzyme levels [113]. Several clinical studies on the treatment of lichen planus have reported enhanced drug efficacy when used in combination therapy, as evidenced by positive treatment outcomes and a shortened time to complete erosion epithelization [110,114,115]. Additionally, Tizol^®^ has been employed in the treatment of moderate chronic generalized periodontitis when combined with Polycatan^®^ [116].

Currently, technologies for obtaining silicon glycerolates and solvate complexes of silicon and titanium glycerolates are known, and they are also characterized by increased transcutaneous activity [117,118,119]. Zabokrytsky N.A. conducted preclinical studies in which the author examined the pharmacological properties of a gel containing not only the drug and Tizol^®^, but also a silicon-titanium-organic glycerol-hydrogel. The obtained data confirmed the high efficacy of these agents in treating first- and second-degree thermal burns, as evidenced by a more rapid reduction in the affected area and an increase in humoral immunity indicators, including phagocytic activity of blood neutrophils and peritoneal macrophages, as well as the levels of T- and B-lymphocytes, antibody-forming cells, and various classes of immunoglobulins [120]. However, despite the studies confirming the effectiveness of the combination of Tizol^®^ with other drugs, the mechanism remains incompletely understood to this day. The question of improving penetration with glycerin compounds containing heavy metals also remains unresolved. Additionally, researchers have noted the presence of carcinogenic properties in titanium oxide [121], raising concerns regarding the absence of toxicity in Tizol^®^, as claimed by the developers of the compound.

#### 2.3.7. Polymeric Nanoparticles

Polymeric nanoparticles have recently attracted considerable attention in the pharmaceutical industry due to their advantageous properties, including ease of production, high drug-loading capacity, biocompatibility, and biodegradability. These systems consist of a polymer (e.g., polyethylene glycol), a stabilizing agent (e.g., polyvinyl alcohol), and a therapeutic agent. There are six types of polymer nanoparticles: (1) nanospheres and nanocapsules, (2) micelles, (3) dendrimers, (4) polymersomes, (5) polyelectrolyte complexes, and (6) hybrid polymer systems [122]. Nanospheres are solid colloidal particles (10–1000 nm) where drug molecules are dispersed in a polymer matrix, while nanocapsules consist of a liquid or solid core surrounded by a polymeric shell—both classified under polymeric nanoparticles for drug delivery. The work of Varela-Fernández et al. reports the design and development of Lactoferrin-loaded poly (lactic-co-glycolic) acid (PLGA) nanoparticles using a three-component mixture of drug/polymer/surfactant (Lf/PLGA/Poloxamer), modified by nanoprecipitation methods. The results confirmed the controlled release of the drug, making this drug delivery system optimal for topical ophthalmic delivery [123].

Polymeric micelles are self-assembled colloidal structures (10–100 nm) formed by amphiphilic block copolymers that create a hydrophobic core and hydrophilic corona, useful for encapsulating and delivering hydrophobic drugs. A study by Brunato et al. focused on the design of methoxypolyethylene glycol (mPEG)-polyaminoacid-based micelles, for controlled release of the drug doxorubicin [124].

Dendrimers are branched, highly monodisperse synthetic macromolecules characterized by a globular, tree-like structure. Essien et al. developed a phenylboronic acid-PAMAM dendrimer (PBA-G5D), which is loaded with Methotrexate and sulforaphane, designed as a combination therapy for anti-inflammatory effects in rheumatoid arthritis [125].

Polymersomes are synthetic structures formed from amphiphilic block copolymers that self-assemble into vesicular architectures. Xu et al. demonstrated a facilitated method to encapsulate anti-cancer drug paclitaxel (PTX) into PEG-b-PCL-based polymersomes (PTX@PS) [126].

Polyelectrolyte complexes are spontaneously formed nano-carriers created by electrostatic interaction between oppositely charged polyelectrolytes. Unagolla et al. developed chitosan/alginate polyelectrolyte complex (PEC)-based microparticles via the ionotropic gelation method for the systemic delivery of vancomycin. Compared to microparticles formulated with either polymer alone, the lyophilized PEC microparticles demonstrated better control over drug release, maintaining a rate of 22 μm/day for 14 days [127].

Hybrid polymer nanoparticles consist of a polymeric core surrounded by a lipid or phospholipid shell, integrating the advantages of both materials to enhance stability and drug delivery performance. Tuğcu-Demiröz et al. developed a hybrid nanofiber system incorporating chitosan nanoparticles for the controlled vaginal release of benzydamine. While gel formulations had better mucoadhesive properties, the nanoparticle-loaded nanofibers achieved greater drug penetration through vaginal tissue [128].

#### 2.3.8. Solid Lipid Nanoparticles (SLNs)

Solid lipid nanoparticles are promising nanocarriers consisting of a solid lipid core stabilized by emulsifiers. It is believed that after applying a formulation with SLNs, an occlusive film is formed, which reduces transepidermal water loss and increases skin hydration, thereby improving the penetration of the encapsulated active substances within the SLNs [129]. SLNs were developed in the early 1990s as an alternative to emulsions, liposomes, and polymeric nanoparticles [130]. The advantages of SLNs include reduced mobility of encapsulated active substances, prevention of particle aggregation, and sustained release of the active ingredient. Since the main matrix of SLNs is solid lipid, their affinity for lipophilic drugs is greater than for hydrophilic ones. However, some methods allow the application of SLNs for hydrophilic drugs. These methods include coating the surface of SLNs with biocompatible polymers (e.g., chitosan or hyaluronic acid) and polyelectrolyte multilayering [131]. SLNs outperform other colloidal systems in terms of physical stability, duration of action of active substances, and high biocompatibility. However, the ideal lipid crystals in SLNs limit the solubility of the drug, which leads to displacement phenomena, insufficient loading of active substances, and lower nanoparticle concentration [131]

#### 2.3.9. Nanostructured Lipid Carriers (NLCs)

Nanostructured lipid carriers were developed to overcome the limitations of SLNs [129]. NLCs are formulated using solid and liquid lipids, which serve to enhance drug loading, release profiles, and storage stability of active substances [132]. The advantages of NLCs include the protection of encapsulated drugs from chemical degradation, controlled release of active substances, and increased absorption. Like SLNs, NLCs can penetrate the stratum corneum by forming a film on the skin’s surface. Additional mechanisms contributing to enhanced permeability may include lipid rearrangement in the stratum corneum, mixing of NLC lipids with stratum corneum lipids via surfactants, and widening of gaps between corneocytes [133]. NLCs loaded with agomelatine have been developed and encapsulated in a carbopol hydrogel for greater stability. The permeability of this formulation was compared to that of an agomelatine suspension gel, with the NLC formulation exhibiting a 2.1 times greater penetration rate than the suspension gel [134]. Another study developed a transdermal patch with capsaicin encapsulated in NLCs. When applied to the skin, capsaicin can cause undesirable effects such as burning, erythema, and skin irritation, leading to low patient adherence to treatment. These side effects are attributed to the rapid penetration of capsaicin into the outer layer of the epidermis and its limited penetration into the dermis. NLCs allow for the delivery of capsaicin to deeper layers of the skin compared to conventional capsaicin patches. In vivo skin irritation studies demonstrated that capsaicin-loaded NLCs induced fewer adverse effects compared to conventional capsaicin patches [135]. An interesting approach involves the coating of NLCs loaded with tetrahydrocurcumin with chitosan for breast cancer treatment. This formulation has been reported to enhance skin permeability, cellular uptake, and cytotoxicity of tetrahydrocurcumin in vitro [136].

#### 2.3.10. Self-Emulsifying Systems

Self-emulsifying systems are isotropic mixtures of an active compound combined with lipids, surfactants, and hydrophilic co-solvents or solubilizers. These systems spontaneously form emulsions upon moderate mixing in an aqueous phase [137]. They exhibit a high solubilizing capacity for lipophilic compounds, possess thermodynamic stability, are easy to manufacture, and offer an aesthetically pleasing appearance. Upon local application, self-emulsification followed by the formation of an occlusive system occurs when the aqueous phase reaches the skin surface due to sweat secretion or transepidermal water loss [138]. Hu et al. developed a self-double-emulsifying system with resveratrol for transdermal delivery, using evening primrose oil as the lipid phase. The final composition consisted of a mixture of hydrophilic surfactant (Tween 60) with an oil-in-oil (o/o) emulsion. The developed self-double-emulsifying system demonstrated increased skin accumulation and permeation compared to an aqueous solution of trans-resveratrol in in vitro testing [139]. El Maghraby (2010) compared the transdermal delivery of the model substance indomethacin using self-microemulsifying systems and microemulsions. The self-microemulsifying system contained ethyl oleate as the oily phase and Tween 80/Span 20 as surfactants. In vitro skin penetration studies demonstrated that the self-emulsifying system, when used in an occlusive application, significantly increased the penetration rate compared to other formulations [140]. Badran et al. (2014) investigated the influence of surfactant type on the transdermal delivery of meloxicam using ultra-thin self-nanoemulsifying systems. A series of formulations with varying surfactants was prepared for this purpose. The first formulation contained only Cremophor RH 40; the second contained Cremophor RH 40 and Tween 60; the third contained Cremophor RH 40 and Capmul MCM C8; the fourth contained Cremophor RH 40 and PEG400; the fifth contained Cremophor RH 40, Tween 60, Capmul MCM C8, and PEG 400. In vitro investigation of transdermal delivery revealed that the fifth formulation exhibited the highest values for transdermal penetration parameters [141].

#### 2.3.11. Exosomes

Exosomes are natural nanoparticles measuring 30–150 nm in diameter that are released into the extracellular space following the fusion of multivesicular bodies with the plasma membrane [142]. They participate in intercellular communication by penetrating target cells via endocytosis, pinocytosis, phagocytosis, or membrane fusion [142].

Unlike synthetic carriers, plant exosome-like vesicles (PELNVs) perform not only a transport function, but also a therapeutic function, due to their high stability, biocompatibility, penetrating ability, low immunogenicity, and inherent biological activity.

Exosomes penetrate the skin in two stages: first, they overcome the stratum corneum via intercellular and transdermal routes; then, they undergo cellular uptake via endocytosis and phagocytosis. Experimental data show that PELNVs, when isolated from sources such as broccoli, cucumber, beetroot, and wheat, can enhance the delivery of lipophilic compounds to the dermis [143,144]. They can also stimulate the proliferation and migration of fibroblasts and endothelial cells (HUVEC), activate angiogenesis, and increase collagen I and III synthesis. Furthermore, grapefruit exosomes have been shown to reduce intracellular reactive oxygen species (ROS) levels, increase HaCaT cell viability, and promote the formation of capillary-like structures in HUVEC cultures in a dose-dependent manner [145].

Exosomes produced by mesenchymal stem cells are of particular interest. Exosomes obtained from adipocyte stem cells and modified by the METTL3 methyltransferase have demonstrated the ability to stimulate the proliferation and migration of dermal fibroblasts. This effect is mediated by m^6^A modification of the CCNB1 mRNA, which is involved in cell cycle regulation. This confirms the regenerative potential of such vesicles [146].

To improve the efficiency of transdermal delivery, it is possible to encapsulate exosomes in microneedles, which create microchannels in the stratum corneum [147]. This system ensures precise delivery of the exosomes to the epidermis and dermis, where they activate skin cells and enhance regeneration.

Therefore, a combination of plant- and cell-derived exosomes with physical delivery methods forms an effective and safe platform for the transdermal administration of therapeutic agents.

The characteristics of these nanocarrier systems are compared in Table 5.

A more in-depth discussion of other rarer nanocarriers can be found in reviews on nanosystems. In the case that the utilization of a nanocarrier is feasible, the selection of the nanocarrier can be made based on the properties of the active ingredients and the dosage form [148,149].

Table 6 summarizes the possible strategies for permeability enhancement. In addition, they may be combined when required.

## 3. Assessment Methods for Transdermal Drug Delivery Systems

### 3.1. In Vitro/Ex Vivo Methods

#### 3.1.1. Vertical Diffusion Cells (VDCs)

The vertical diffusion cell (VDC), also known as the Franz diffusion cell, has been widely used to analyze transdermal and topical dosage forms for both in vitro permeation test (IVPT) and in vitro release test (IVRT) studies [151,152,153,154]. The cell consists of a donor and a receptor compartment (chamber), between which a membrane is fixed and secured with a joint clamp and O-ring. The dosage form is placed in the donor compartment. The APIs passing through the membrane enter the receptor solution, and then the sample is withdrawn from the receptor compartment through the sampling port. The cell surrounded by a jacket is to maintain physiological skin temperature. Maintaining a constant temperature (32 ± 1 °C) is essential to ensure the relevance of the study [151,152]. The IVRT study aims to evaluate the release kinetics of active ingredients from a product depending on various variables. Synthetic membranes are commonly used for IVRT studies because they are the most standardized. The receptor solution can be an aqueous buffer for water-soluble drugs or an aqueous/alcoholic medium for poorly water-soluble drugs. A pseudo-infinite dose condition is applied to ensure a stable release rate. The method estimates the release rate (flux) and extent of release (the cumulative amount of APIs released and lag time). The apparent steady-state flux can be measured [154,155,156,157,158,159]. For instance, Lane et al. (2013) reported a steady-state flux of 293.3 ± 23.63 μg/cm^2^/h for ibuprofen administered from a solution of ethanol/water in a ratio of 75/25 through human epidermis using Franz cell experiments with an infinite dose. In contrast, at an ethanol/water ratio of 25/75, the flux was significantly lower, at 48 ± 19.34 μg/cm^2^/h [160]. The IVPT study is more relevant and aims to assess the in vitro percutaneous penetration of active ingredients. The VDC was designed originally to examine in vitro percutaneous absorption, and the method itself intends to replicate real in vivo conditions. A finite dose is applied, and receptor solutions that closely resemble physiological conditions—such as aqueous buffers or solutions containing surfactants—are used. Either human or animal skin may be employed [157,159,161]. The main parameter studied is the cumulative amount of drug permeated or absorbed. Maintaining and controlling skin temperature and receptor environment is critical for IVPT results. Since skin is used for IVPT studies, it is essential to determine the integrity of the skin barrier, which is described further below. Although IVPT is commonly used to determine the bioequivalence of topical products, it can also be applied during the pharmaceutical development stage to compare enhancers [160,162]. Successfully conducted laboratory studies can identify potential problems early in development and avoid potential failures in clinical trials. For example, to study the effect of compositional changes on API penetration [163,164]. Such a study contributes to a more efficient and cost-effective drug development process. While many sources emphasize the low cost of the method compared to conducting clinical trials on topical agents, there are still several challenges related to the availability and cost of human skin, which is needed in large quantities and from a significant number of donors, assessment of skin barrier integrity, variability of results, risk of skin damage, aqueous boundary layer (ABL), qualification of personnel to conduct the study [152,165].

#### 3.1.2. Flow Cells

Flow cells were introduced in 1984. Unlike static diffusion cells, the acceptor medium in flow cells is continuously circulated using a peristaltic pump. This dynamic flow simulates skin blood circulation, which removes exogenous compounds absorbed through the skin, thereby providing a closer approximation to in vivo conditions and greater reliability than static systems. Furthermore, flow cells can be equipped with auto-sampling mechanisms, reducing labor-intensive procedures and enabling continuous monitoring of absorption profiles.

Despite these advantages, static Franz diffusion cells remain more commonly used in transdermal drug delivery studies due to the greater complexity and cost associated with flow cell systems [166,167].

#### 3.1.3. Organ-on-a-Chip Devices

Organ-on-a-chip devices are microfluidic devices with micrometer-sized chambers that allow the cell culture to simulate the physiology of tissues or organs [168]. Traditional Franz diffusion cells are expensive, have low throughput, and suffer from poor reproducibility [169]. Skin-on-a-chip devices are innovative platforms that address the limitations of diffusion cell methods [170]. The skin-on-a-chip platform enables the use of three-dimensional skin models that replicate the structures of the epidermis and dermis [171]. In 2019, Lukács et al. developed a microfluidic diffusion chamber device with several advantages, including low cost, minimal drug and skin consumption, small sample volumes, dynamic positioning with continuous flow mimicking skin circulation, and fast, reproducible results [172]. J. Kim et al. (2020) used a pump-free skin-on-a-chip model in a systematic study of the efficacy of coenzyme Q10. This research group noted that, with this technology, the drug efficacy is analyzed histologically and molecularly, as the epidermis and dermis are cultured together, unlike in 2D skin models, thereby increasing the biological relevance of the method [173]. K. Kim et al. (2020) evaluated the platform’s effectiveness by testing the efficacy of *C. longa* leaf extract using the skin-on-a-chip platform [174]. Skin-on-a-chip could become a valuable in vitro model for representing interactions between drugs and skin tissues, as well as a realistic platform for assessing skin responses to pharmaceutical materials and cosmetic products [175].

#### 3.1.4. Tape Stripping

Tape stripping is widely used to determine the amount of drug substance absorbed into the stratum corneum. The core principle of this method involves the repeated removal of the stratum corneum using pre-prepared adhesive tapes. In their experiment, Jonsdottir et al. (2022) applied and removed adhesive tape from each skin sample a total of 70 times. They then performed extraction with methanol from both the adhesive tapes and the remaining skin with subcutaneous adipose tissue. Following extraction, the researchers analyzed aliquots of the extract using high-performance liquid chromatography (HPLC) [176].

### 3.2. In Vivo Models

In vivo models provide a more accurate assessment of drug permeation through the skin. These models are based on methods such as dermal open-flow microperfusion (DOFM), microdialysis, and direct measurement of pharmacokinetic and pharmacodynamic parameters of the drug in plasma [177]. Bodenlenz et al. (2017) evaluated dermal open-flow microperfusion to determine the intradermal bioavailability of acyclovir. Their results showed that this method could directly measure the penetration of acyclovir in vivo with low variability over extended periods [178]. Schwagerle et al. (2023) compared pharmacokinetic data from in vitro permeation testing (IVPT) and open-flow microperfusion of acyclovir formulations. The formulations that performed best in IVPT were further analyzed using the open-flow microperfusion method. This experiment revealed differences in the dermal concentration of acyclovir that were not detected by the first method [179]. Shinkai et al. used microdialysis to investigate the amount of ketoprofen released from patches applied to the skin of rats and pigs, which reached the knee joint. The study results indicated that ketoprofen was easily detected in the knee joints at the application site, while in areas where it was not applied, the drug was virtually undetectable, confirming that the drug penetrated via direct diffusion rather than systemic circulation [180]. Fan et al. (2022) compared the transdermal delivery of sinomenine hydrochloride using liposomes and transferosomes. The researchers employed VDCs as an ex vivo method for determining drug penetration and microdialysis as an in vivo method to assess pharmacokinetic parameters. While microdialysis only measures free sinomenine hydrochloride, the in vivo absorption curves correlated well with the ex vivo permeation curves. Based on these data, it was concluded that the pharmacokinetic properties of the delivery systems reflected their ex vivo penetration behavior [181]. Tariot et al. (2022) compared the steady-state pharmacokinetics of transdermal and oral administration of donepezil. To assess pharmacokinetics, the researchers collected blood samples from study participants both before drug administration and at specific time points afterward. The pharmacokinetic profiles of donepezil and its active metabolite were determined in plasma. The study concluded that 10 mg transdermal donepezil administered weekly is bioequivalent to 10 mg oral donepezil tablets taken daily [182].

## 4. Visualization and Readout Methods for Evaluating Skin Penetration

### 4.1. Confocal Laser Scanning Microscopy (CLSM)

Confocal laser scanning microscopy is widely employed to study the degree of drug penetration through the skin and the effects of various permeation enhancers. Additionally, this method allows for tracking the path of the drug after application to the skin [177]. For instance, using CLSM, Abdel-Hafez et al. (2018) demonstrated that transfollicular penetration is a more prominent characteristic of chitosan nanoparticles [183].

### 4.2. Confocal Raman Spectroscopy (CRS)

Confocal Raman spectroscopy is increasingly becoming popular in skin penetration studies due to its high spatial resolution and chemical sensitivity. CRS allows for the acquisition of detailed depth profiles of topically applied formulations, making it an effective tool for both in vivo and ex vivo studies [184]. Dos Santos et al. (2016) examined the behavior of retinyl acetate during transdermal application without using permeation enhancement methods via CRS. The spectroscopic data revealed that retinyl acetate penetrates the skin to a depth of up to 20 μm [185]. Botelho et al. (2014) evaluated the safety and efficacy of transdermal progesterone with estrogen delivery. CRS enabled the determination of drug penetration depth and concentration in the skin at specific time points after application [186].

## 5. Membranes and Skin Models

### 5.1. Human Skin

Human skin is considered the most suitable membrane for assessing transdermal drug delivery of various formulations. Analysis of studies has shown a high correlation between permeation measured in vitro using excised human skin and in vivo data in humans, provided that experimental conditions are harmonized [164]. Sources of human skin include plastic surgeries, amputations, and cadaveric materials. Skin samples are primarily taken from the abdomen, back, legs, or chest [157,159]. The thickness of the skin layer can be controlled, for example, by using a dermatome. In one study, a 0.5 mm-thick layer was used. It is emphasized that skin barrier properties can vary considerably depending on the anatomical site and individual differences between donors, so regulations describe requirements that help select the most standardized sites [157,159]. However, the use of human skin is highly limited due to ethical considerations and the need for specialized laboratory equipment [187].

EMA and FDA guidelines emphasize the importance of verifying skin integrity [157,159]. The skin is easily damaged, and the use of cryoprotectants is limited. Skin barrier integrity is currently assessed using the Transepidermal Water Loss (TEWL), Transcutaneous Electrical Resistance or Impedance, Trans-Epithelial Electrical Resistance assay, and Tritiated Water Permeability tests [188]. Among all the methods, the TEWL method is the most common. For barrier integrity testing, TEWL on human torso or thigh skin is typically no more than 15 g/m^2^ 238/hr, but other acceptable results may be justified in some cases. The skin may be slightly moisturized during the study, but the method should not irreversibly alter the skin barrier [157,159].

### 5.2. Animal Skin

Animal skin can be used in cases where access to human skin is limited. Pig skin is recognized as the most suitable animal model due to its numerous anatomical, histological, and physiological similarities with human skin, including (1) epidermal thickness, (2) the ratio of epidermal to dermal thickness, (3) hair follicles, (4) vascular density, (5) the content of glycosphingolipids in the stratum corneum, ceramides, dermal collagen, and elastin. In addition to pig skin, the skin of other animals—such as primates, mice, rats, guinea pigs, rabbits, cattle, and snakes—is also used. Because research on primates is highly regulated and expensive, rodent skin can serve as an alternative model for both in vitro and in vivo transdermal studies. Unlike pig skin, the use of rodent skin requires ethical approval. The advantages of rodents include their small size, ease of handling, and relatively low cost. Additionally, several hairless species, such as nude mice, hairless rats, and guinea pigs, lack fur, making their skin a closer analog for human skin [187]. Despite these benefits, regulatory acceptance of animal skin varies. Some countries allow its use in permeability studies for determining bioequivalence, while others do not accept it at all [189]. 

It is important to note that the results of tests used to assess skin barrier integrity, such as TEWL, may vary for animal models.

### 5.3. Artificial Membrane

In light of the aforementioned drawbacks of using human and animal skin, significant attention has been drawn to an alternative approach, namely the use of artificial membranes composed of polysulfone (PSU), polyethersulfone (PES), cellulose, or phospholipid vesicles. In vitro permeability data obtained from these systems tend to be less variable due to the absence of anatomical differences, making artificial membranes particularly useful for studying drug release mechanisms. Examples of such membranes include Strat-M^®^ (Merck) and Skin-PAMPA [177,190].

### 5.4. Human Skin Equivalents

Human skin equivalents are bioengineered three-dimensional constructs made up of cultured skin cells. These models can be classified into the reconstructed human epidermis (RHE) and full-thickness systems (FT), which contain both the epidermis and dermis. Examples of commercially available RHE include EpiDerm™, Episkin™, and SkinEthic™, while FT is commercially available under brands such as Epi-DermFT^®^, Vitrolife-Skin™, GraftSkin^®^, etc. [191].

#### 5.4.1. MIVO^®^ (Multi In Vitro Organ) Systems

Multi in Vitro organ systems are disposable chambers for cell culture designed to accommodate live tissues or artificial membranes under physiological conditions. They enable multiple fluid circulations, simulating the human circulatory system with vascularization of the tissue of interest. In 2022, Pulsoni et al. designed an MIVO system compliant with OECD 428. Like Franz cells, this system consists of donor and receptor chambers separated by a membrane. The acceptor chamber is designed to connect to a peristaltic pump. The system also includes a three-way valve, allowing for sampling without affecting the sterility of the surrounding environment and tissue. Their study showed that the MIVO^®^ system and Franz diffusion cells presented comparable kinetic profiles for caffeine and LIP1 across Strat-M^®^ membranes and pig skin. Interestingly, LIP1 penetration into Strat-M^®^ with MIVO^®^ was 17.6% that although with pig skin, it was 5.4%, supporting that the system can reproduce the human permeability for lipophilic substances and presenting physiological relevance when compared to in vivo conditions [167].

#### 5.4.2. Skin-PAMPA (Parallel Artificial Membrane Permeability Assay) System

Skin-PAMPA is a sandwich-structured model consisting of donor and acceptor chambers separated by a membrane, integrated into a 96-well plate [190,192,193]. The membrane in skin-PAMPA mimics key aspects of the skin’s composition, including free fatty acids, cholesterol, and synthetic ceramide analogs, in comparable proportions [169,190]. Sinkó et al. (2021) successfully used skin-PAMPA as a screening tool to compare the permeability of the model compound 4-phenylethyl-resorcinol dissolved in various solvents [194]. Rahma et al. (2023) demonstrated strong correlations for 2-phenoxyethanol penetration between PAMPA skin and mammalian skin, with r^2^ values of 0.84 (porcine) and 0.89 (human) under finite dose conditions. These results confirm the model’s ability to reflect real-world topical exposure scenarios and its potential usefulness in early-stage screening [193]. Similarly, Zsikó et al. (2020) noted that the skin-PAMPA method could potentially serve as a selection tool for identifying the best dermal formulation in the pharmaceutical, cosmetic, and personal care industries [193,195].

## 6. Regulatory Aspects of Transdermal Drug Delivery Systems

The development, evaluation, and approval of TDDs are carefully regulated by authorities like other medicines. Compared to other remedies, they are a less traditional dosage form. Even generics of TDDs are classified as “complex generics”. In addition to national and international pharmacopoeial and registration requirements, some agencies, such as the US Food and Drug Administration (FDA) and the European Medicines Agency (EMA), have developed specific recommendations regarding the quality, safety, efficacy, and bioequivalence of TDDs.

### 6.1. Regulatory and Safety Framework for Transdermal Systems

Approval of TDDs requires adherence to good laboratory practices (GLP) to ensure quality, reproducibility, and traceability of data. Drugs must also meet safety standards for topical dermal toxicity, sensitization, phototoxicity, and systemic exposure. In addition, TDDs that include device-like components (e.g., micro-needles, iontophoresis devices) are regulated as combination products. This involves adherence to both pharmaceutical and device-specific standards, including labeling, biocompatibility testing [196], and risk management [197]. Global harmonization efforts, led by organizations such as ICH and OECD, support the integration of innovative assessment platforms such as skin-PAMPA and organ-on-chip models, subject to sufficient validation and regulatory approval.

### 6.2. Bioequivalence Assessment of Transdermal Drug Delivery Systems

Bioequivalence of transdermal systems is typically evaluated through in vivo PK studies using a single-dose, two-period crossover design. Multiple-dose studies may be required if accumulation is expected. Key PK parameters include Cmax, AUC_0−t_, and AUC_0−∞_, with 90% CI for test/reference ratio within 80–125%. Studies are conducted in healthy adults under standardized conditions, with controlled diet and activity. Frequent blood sampling near Tmax and over ≥3 half-lives is required. Adhesion is assessed separately using a 5-point scale, etc. The highest score is carried forward; non-inferiority is shown with a margin of 0.15. Local tolerability (irritation, sensitization) must not exceed that of the reference. Studies often follow a 21-day induction and challenge protocol. Both FDA and EAEU require combined evaluation of PK, adhesion, and safety to ensure equivalence of TDDS.

EMA and the Eurasian Economic Commission (EEC) also accept such studies under harmonized protocols and EEC Decision No. 85 [158,159].

### 6.3. IVRT and IVPT

Currently, IVRT and IVPT guidelines have been developed and are used to assess the bioequivalence of topical but not transdermal agents. This is due to the fact that APIs from transdermal agents are directed to reach the systemic bloodstream. Nevertheless, standardized methodologies can be used in early phase studies, the requirements for assessing the integrity of the skin barrier, which are described in guidelines from the FDA, EMA, EEC, etc. [157,158,159].

Such requirements include, for example, transepidermal water loss or electrical resistance values for extracted human skin, as well as skin thickness, characteristics of the excised skin, freezing, and storage conditions.

## 7. Conclusions

The active development of transdermal drug delivery systems in the pharmaceutical industry continues to advance. These innovative systems offer an alternative method of drug administration that is not readily achievable by conventional routes. However, the success of this approach depends on the physicochemical properties of the drug—such as molecular weight and lipophilicity—as well as the strategies employed to overcome skin barriers. In this review, we examined various methods to enhance skin permeability.

Active physical methods, including iontophoresis, sonophoresis, and microneedles, represent promising approaches for increasing transdermal drug penetration. Additionally, the development of vesicles and nanocarriers through passive methods has shown potential in improving drug transport across the skin. Microemulsions, known for their ability to dissolve both lipophilic and hydrophilic drugs, as well as their excellent thermodynamic stability and enhanced drug permeability, are widely used in transdermal delivery systems. To overcome the stability issues associated with microemulsions, researchers have focused on using biocompatible and stable solid lipid nanoparticles (SLNs). Furthermore, the development of nanostructured lipid carriers (NLCs) has addressed challenges related to drug release during storage. Invasomes, glycerosomes, and transferosomes—nanocarriers characterized by high deformability and flexibility—are also under active investigation due to their potential for targeted and efficient drug delivery. Importantly, traditional chemical methods for enhancing skin permeability should not be overlooked. Penetration enhancers are used both alone and in combination with physical or nanotechnology methods.

Evaluating the effectiveness of transdermal drug delivery systems is also crucial in the pharmaceutical industry. We reviewed various analytical methods, such as in vitro, ex vivo, and in vivo assessments, for evaluating drug penetration through the skin. To ensure a more comprehensive and accurate evaluation of transdermal drug delivery systems, it is recommended to use a combination of these methods.

## Figures and Tables

**Table 1:** Influence of physicochemical factors on transdermal drug delivery.

Parameter	Limits
Water solubility	>1 mg/mL
Lipophilicity (P_o/w_)	10–1000
Molecular weight	<500 Da
Melting temperature	<200 °C
pH of saturated water solution	5–9
Dose	<10 mg/day

**Table 5:** Nanocarrier Systems.

Carrier Type	Key Features	Advantages	Disadvantage	Reference
Nanoemulsions	Submicron o/w, w/o, or bicontinuous systems; stabilized by surfactants; nanometer droplet size	High drug solubilization; enhanced skin permeation; encapsulates both hydrophilic and lipophilic drugs; kinetic stability	High surfactant content (>20%) can irritate skin; surfactant reduction needed	[36,66,71]
Microemulsions	Transparent, thermodynamically stable systems; droplet sizes typically below 0.1 μm (100 nm); surfactant-stabilized o/w or w/o phases	Excellent solvent capacity; increased permeability; easy to prepare; surfactant and lipid-based skin disruption	High surfactant content (>20%) may cause skin irritation	[66,67,68,70]
Liposomes	Spherical vesicles with phospholipid bilayers; encapsulate hydrophilic, lipophilic, and amphiphilic drugs	Versatile drug loading; biocompatible; modifiable with PEG, ligands for targeting	Short half-life, rapid clearance, instability in plasma	[72,73,77]
Invasomes	Modified liposomes with phospholipids, ethanol, and terpenes; enhance penetration via synergistic effects	Improved drug penetration; utilize natural enhancers; effective for various APIs	Potential skin irritation due to terpene and ethanol content	[81,82,83]
Transferosomes	Highly elastic, ultra-deformable vesicles with phospholipids, surfactants, and ethanol	Enhanced skin penetration through deformation; suitable for diverse solubility drugs	Requires surfactants	[84,85]
Ethosomes	Transferosomes with high ethanol content; ethanol disrupts lipid packing in the stratum corneum	Deep skin layer penetration; improved delivery via vesicle fusion with membranes	High ethanol content may cause dryness and irritation	[86]
Glycerosomes	Flexible vesicles with 10–30% glycerol; high encapsulation efficiency and deformability	High stability; moisturizing effect; effective for dermal and mucosal delivery	Too much glycerol reduces homogeneity and encapsulation efficiency	[88,90,91,93]
Polymeric Nanoparticles	Composed of polymers (e.g., PLGA, PEG); includes nanospheres, micelles, dendrimers, etc.	Controlled release; high drug loading; biocompatible; diverse structural options	Scaling complexity, potential polymer toxicity, costly synthesis	[122,123,124,131]
Solid Lipid Nanoparticles (SLNs)	Solid lipid matrix stabilized by surfactants; forms occlusive film enhancing hydration	Enhanced skin hydration; sustained release; physical stability; biocompatibility	Limited loading of hydrophilic drugs, potential stability issues	[129,131]
Nanostructured Lipid Carriers (NLCs)	Blend of solid and liquid lipids; overcomes SLN limitations, improves drug loading and stability	Higher drug loading; improved stability; controlled release; deeper skin penetration	Risk of crystallization; production complexity	[132,133,134]
Self-Emulsifying Systems	Isotropic mixtures forming emulsions upon contact with aqueous phase; high solubilizing capacity	Ease of formulation; increased solubility; spontaneous emulsification; occlusive action	High surfactant content may cause irritation; needs optimization	[137,138,140]
Exosomes	Extracellular vesicles (30–150 nm); mediate intercellular communication; penetrate skin via endocytosis, phagocytosis, or membrane fusion	Biocompatibility, low immunogenicity, high skin permeability	Challenges in standardization	[142,143,144,145,146,147]

**Table 6:** Summary of the Enhancement Strategies.

Strategy Type	Examples	Mechanism	Key Features	Reference
Chemical	Ethanol, fatty acids, terpenes, sulfoxides	Disrupt lipid bilayers, increase hydration	Low or moderate cost of production, difficult to use and register the drug	[26,27,31,34,46,47,48,49]
Physical	Microneedles, iontophoresis, sonophoresis, electroporation	Create microchannels or drive drug using energy	High cost of production, difficult to use and register the drug, often refers to a combination of a medical device and a medicinal product	[16,17,54,57,58,59,60,64,150]
Nanocarrier-based	Liposomes, ethosomes, transferosomes, SLNs, NLCs	Enhance solubility, flexibility, and targeting	Moderate cost of production, difficult to use and register the drug	[72,73,84,85,86,129,131,132,133]

## Abbreviations

The following abbreviations are used in this manuscript:

TDD	Transdermal drug delivery
NSAI	Non-steroidal aromatase inhibitor
APIs	Active pharmaceutical ingredients
OLZ	Olanzapine
PEK	Perilla-ketone
DOTAR	1,2-dioleoyloxy-3-trimethylammoniumpropan
ATRA	All-trans retinoic acid
PA	Phosphatidic acid
PEA	Phosphatidylethanol
PC	Phosphatidylcholine
PG	Phosphatidylglycerol
PEG	Polyethylene glycol
PI	Phosphatidylinositol
PS	Phosphatidylserine
PDI	Polydispersity index
PLGA	Poly (lactic-co-glycolic) acid
mPEG	Methoxypolyethylene glycol
PBA-G5D	Phenylboronic acid-PAMAM dendrimer
PTX	Paclitaxel
PEC	Polyelectrolyte complex
SLNs	Solid Lipid Nanoparticles
NLCs	Nanostructured Lipid Carriers
PELNVs	Plant exosome-like vesicles
ROS	Reactive oxygen species
VDCs	Vertical Diffusion Cells
MIVO^®^	Multi In Vitro Organ
PAMPA	Parallel Artificial Membrane Permeability Assay
PSU	Polysulfone
PES	Polyethersulfone
RHE	Reconstructed human epidermis
FT	Full-thickness
HPLC	High-Performance Liquid Chromatography
CLSM	Confocal Laser Scanning Microscopy
CRS	Confocal Raman Spectroscopy
DOFM	Dermal Open-Flow Microperfusion
Cmax	Maximum plasma concentration
AUC	Area Under the Curve
AUC_0−t_	Area Under the Curve from time zero to the last measurable concentration
AUC_0−∞_	Area Under the Curve from time zero extrapolated to infinity—gives the complete systemic exposure
FDA	The United States Food and Drug Administration
EAEU	The Eurasian Economic Union
EEU	The Eurasian Economic Union
EMA	The European Medicines Agency
IVRT	In vitro release test
IVPT	In vitro permeation test
